# Supplementary material for: Characterization of Anticancer Effects of the Analogs of DJ4, a Novel Selective Inhibitor of ROCK and MRCK Kinases
Source: Pharmaceuticals (Basel). 2023 Jul 26;16(8):1060. doi: 10.3390/ph16081060 (PMC10458458; doi:10.3390/ph16081060)
Supplement: Supplementary file 1 [file pharmaceuticals-16-01060-s001.zip › pharmaceuticals-2481275-supplementary.pdf]

Supplementary Figures

Figure S1

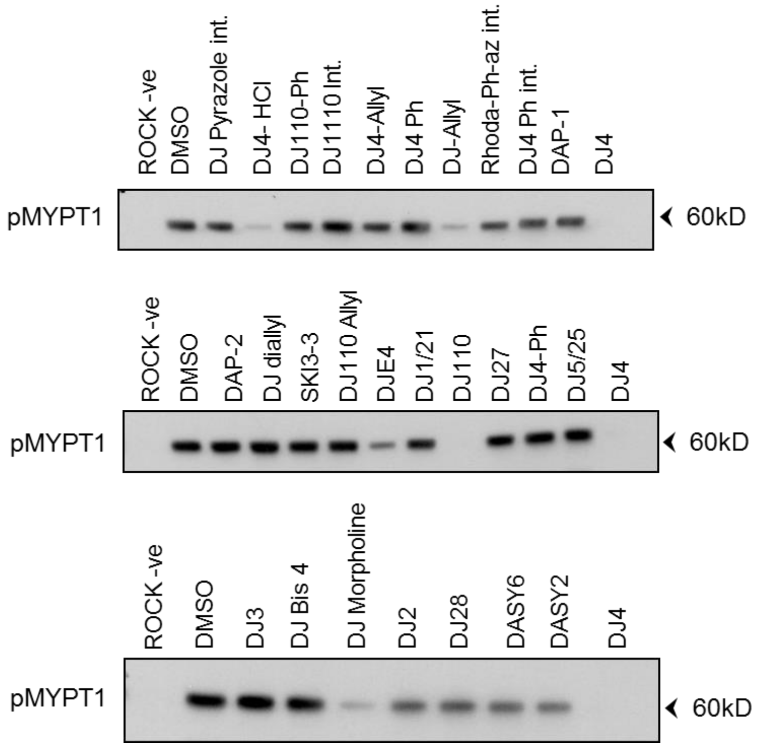

Figure S1. Screening of analogs in the in vitro kinase activity assay to identify active ROCK1 inhibitors
Kinase inhibitory activity of the 27 compounds was evaluated by *in vitro* cell-free biochemical kinase activity assay. The compounds (1 µM) were incubated with recombinant ROCK1 (9.48nM), recombinant MYPT1 (a kinase substrate, 84 nM) and ATP (25 µM). Phosphorylation status of the substrate (pMYPT1) was analyzed to study the kinase activity.

Supplementary Figure S2
A. Single-dose (10μM) NCI60 cell line screen (DJ4)

Panel/Cell Line	Growth Percent
**Leukemia**	
CCRF-CEM	9.93
HL-60(TB)	6.77
K-562	29.92
MOLT-4	11.38
RPMI-8226	39.22
SR	5.60
**Non-Small Cell Lung Cancer**	
A549/ATCC	19.06
EKVX	46.05
HOP-62	19.88
HOP-92	-9.73
NCI-H226	3.30
NCI-H23	9.10
NCI-H322M	5.21
NCI-H460	14.09
NCI-H522	26.62
**Colon Cancer**	
COLO 205	4.78
HCC-2998	53.94
HCT-116	9.69
HCT-15	36.49
HT29	30.57
KM12	13.14
SW-620	9.88
**CNS Cancer**	
SF-268	10.43
SF-295	22.60
SF-539	0.40
SNB-75	-10.87
U251	15.78
**Melanoma**	
LOX IMVI	16.27
MALME-3M	30.65
M14	29.89
MDA-MB-435	20.97
SK-MEL-2	28.79
SK-MEL-28	15.95
SK-MEL-5	14.55
UACC-257	14.56
UACC-62	-1.44
**Ovarian Cancer**	
IGROV1	14.05
OVCAR-3	5.65
OVCAR-4	23.81
OVCAR-5	37.76
OVCAR-8	13.97
NCI/ADR-RES	72.93
SK-OV-3	27.05
**Renal Cancer**	
786-0	9.01
A498	-7.55
ACHN	16.00
CAKI-1	37.44
RXF 393	-20.25
SN12C	15.91
TK-10	34.17
UO-31	25.17
**Prostate Cancer**	
PC-3	25.91
DU-145	20.66
**Breast Cancer**	
MCF7	4.00
MDA-MB-231/ATCC	25.45
HS 578T	8.78
BT-549	-7.90
T-47D	2.60
MDA-MB-468	-14.46
Mean	16.50
Delta	36.75
Range	93.18

Mean Growth Percent - Growth Percent

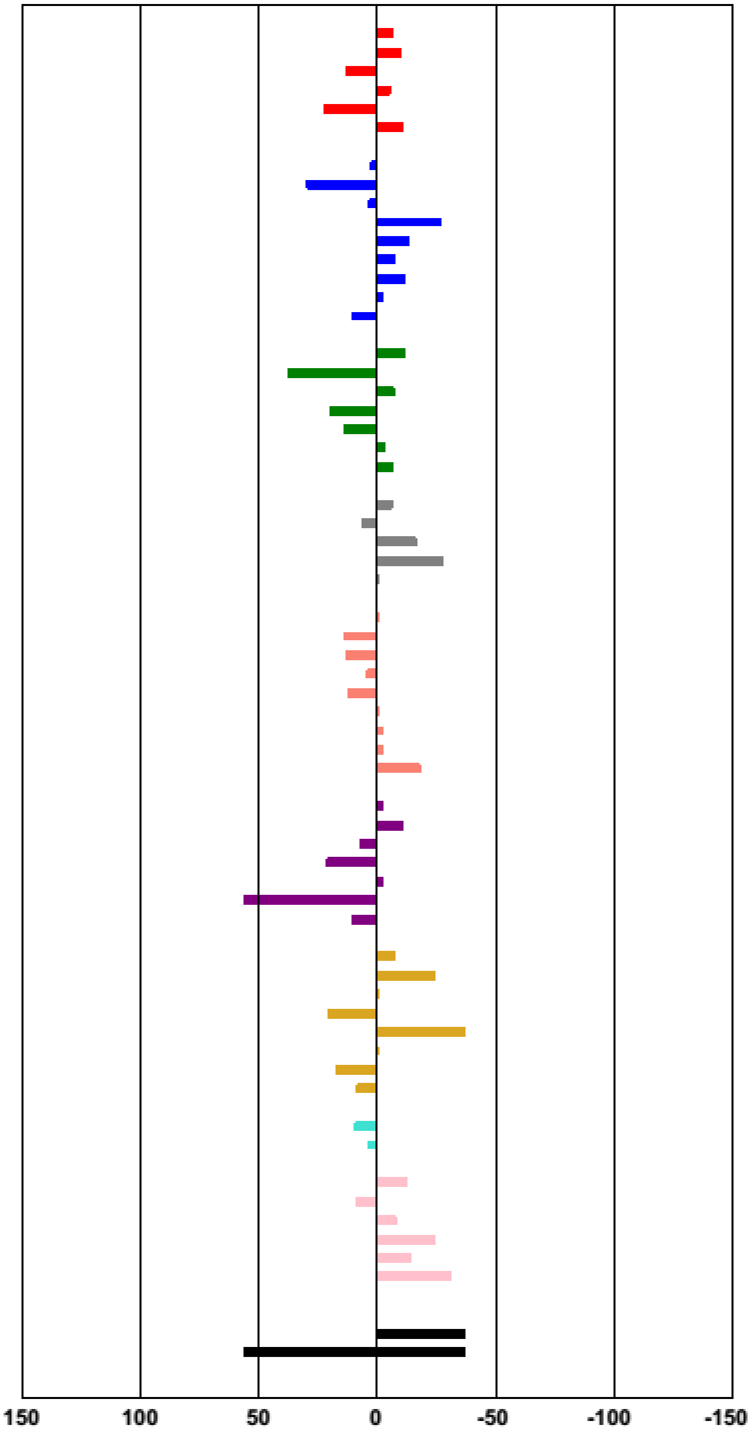

Supplementary Figure S2
B. Single-dose (10µM) NCI60 cell line screen (DJE4)

Panel/Cell Line	Growth Percent
**Leukemia**	
CCRF-CEM	3.01
HL-60(TB)	-22.42
K-562	26.35
MOLT-4	-6.00
RPMI-8226	29.12
SR	0.38
**Non-Small Cell Lung Cancer**	
A549/ATCC	11.11
EKVX	14.06
HOP-62	-26.51
HOP-92	4.42
NCI-H226	10.76
NCI-H23	-7.87
NCI-H322M	5.83
NCI-H460	2.41
NCI-H522	-28.57
**Colon Cancer**	
COLO 205	-33.31
HCC-2998	10.20
HCT-116	3.99
HCT-15	16.04
HT29	4.26
KM12	6.24
SW-620	6.52
**CNS Cancer**	
SF-268	-5.81
SF-295	4.95
SF-539	-64.18
SNB-19	9.99
SNB-75	-71.75
U251	-9.45
**Melanoma**	
LOX IMVI	1.75
MALME-3M	-6.17
M14	0.69
MDA-MB-435	-0.80
SK-MEL-2	-48.69
SK-MEL-28	-17.22
SK-MEL-5	9.15
UACC-257	-15.89
UACC-62	-15.98
**Ovarian Cancer**	
IGROV1	21.66
OVCAR-3	-2.07
OVCAR-4	19.53
OVCAR-5	8.67
OVCAR-8	6.27
NCI/ADR-RES	29.73
SK-OV-3	-10.30
**Renal Cancer**	
786-0	3.02
A498	-54.20
ACHN	0.66
CAKI-1	20.63
RXF 393	-39.33
SN12C	12.56
TK-10	-3.75
UO-31	7.47
**Prostate Cancer**	
PC-3	2.30
DU-145	9.93
**Breast Cancer**	
MCF7	3.97
MDA-MB-231/ATCC	10.67
HS 578T	-15.08
BT-549	-50.36
T-47D	-9.70
MDA-MB-468	-34.72
Mean	-4.36
Delta	67.39
Range	101.48

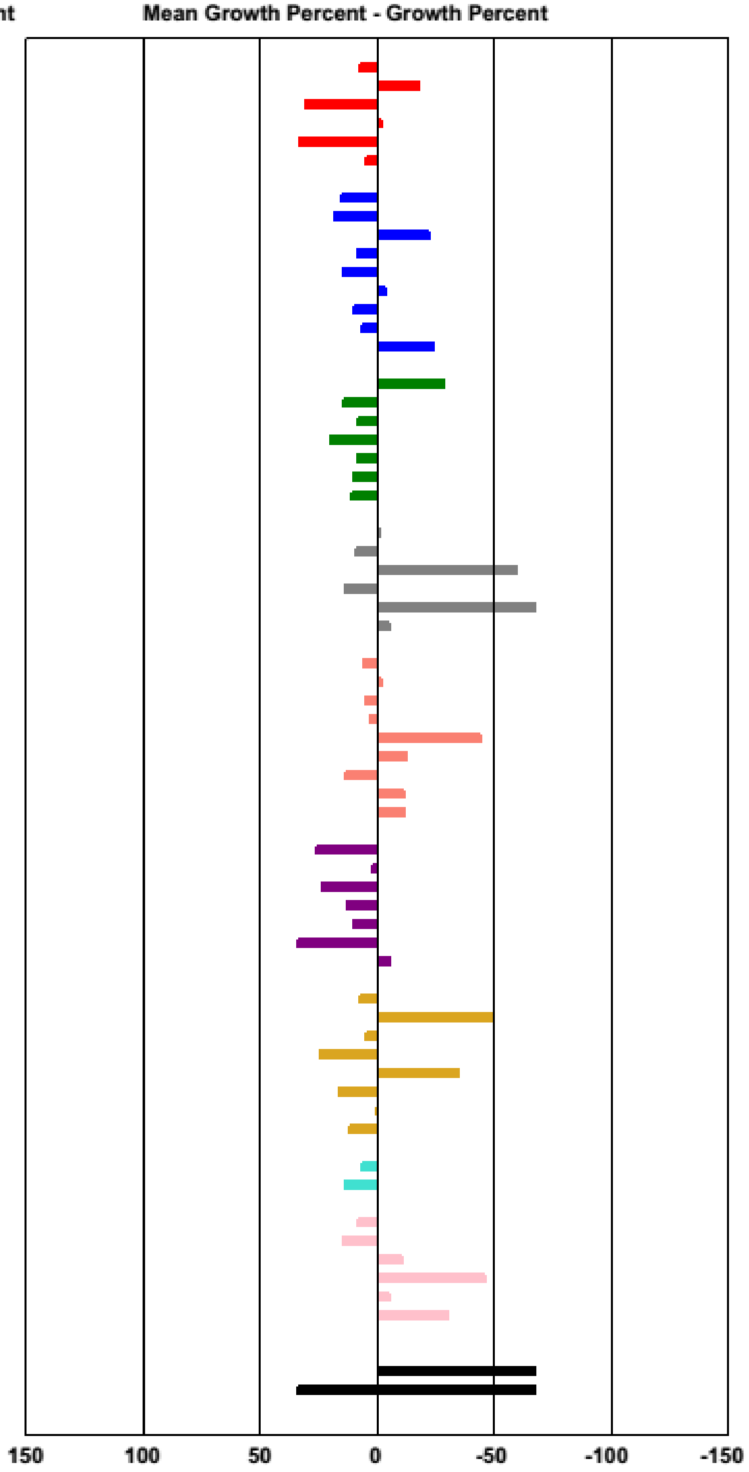

Supplementary Figure S2
C. Single-dose (10µM) NCI60 cell line screen (DJ-Allyl)

Panel/Cell Line	Growth Percent
Leukemia	
CCRF-CEM	7.13
HL-60(TB)	-14.50
K-562	42.06
MOLT-4	5.78
RPMI-8226	42.72
SR	-9.51
Non-Small Cell Lung Cancer	
A549/ATCC	13.25
EKVX	30.48
HOP-62	-10.77
HOP-92	-1.86
NCI-H226	10.69
NCI-H23	6.83
NCI-H322M	10.23
NCI-H460	2.18
NCI-H522	-5.27
Colon Cancer	
COLO 205	-20.09
HCC-2998	44.62
HCT-116	6.31
HCT-15	24.72
HT29	26.36
KM12	7.86
SW-620	7.39
CNS Cancer	
SF-268	-3.26
SF-295	12.98
SF-539	-66.24
SNB-19	11.05
SNB-75	-67.26
U251	0.76
Melanoma	
LOX IMVI	0.55
MALME-3M	40.16
M14	24.51
MDA-MB-435	21.09
SK-MEL-2	-8.35
SK-MEL-28	-2.40
SK-MEL-5	2.01
UACC-257	9.55
UACC-62	9.75
Ovarian Cancer	
IGROV1	17.03
OVCAR-3	-7.02
OVCAR-4	34.72
OVCAR-5	5.27
OVCAR-8	10.74
NCI/ADR-RES	42.09
SK-OV-3	-7.00
Renal Cancer	
786-0	4.40
A498	-34.52
ACHN	-1.22
CAKI-1	30.19
RXF 393	-41.28
SN12C	23.96
TK-10	1.67
UO-31	3.09
Prostate Cancer	
PC-3	12.59
DU-145	12.70
Breast Cancer	
MCF7	3.50
MDA-MB-231/ATCC	19.80
HS 578T	-14.99
BT-549	-29.53
T-47D	-8.68
MDA-MB-468	1.02
Mean	4.83
Delta	72.09
Range	111.88

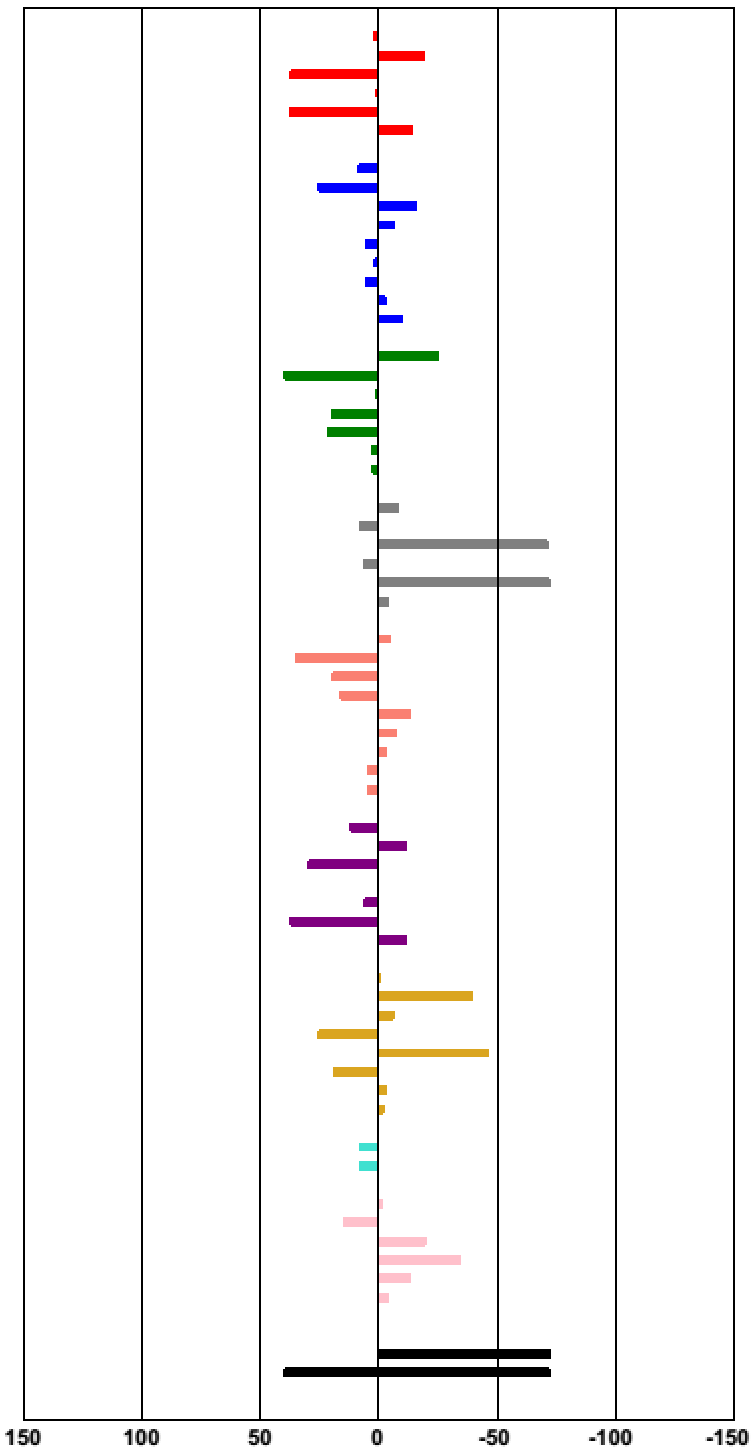

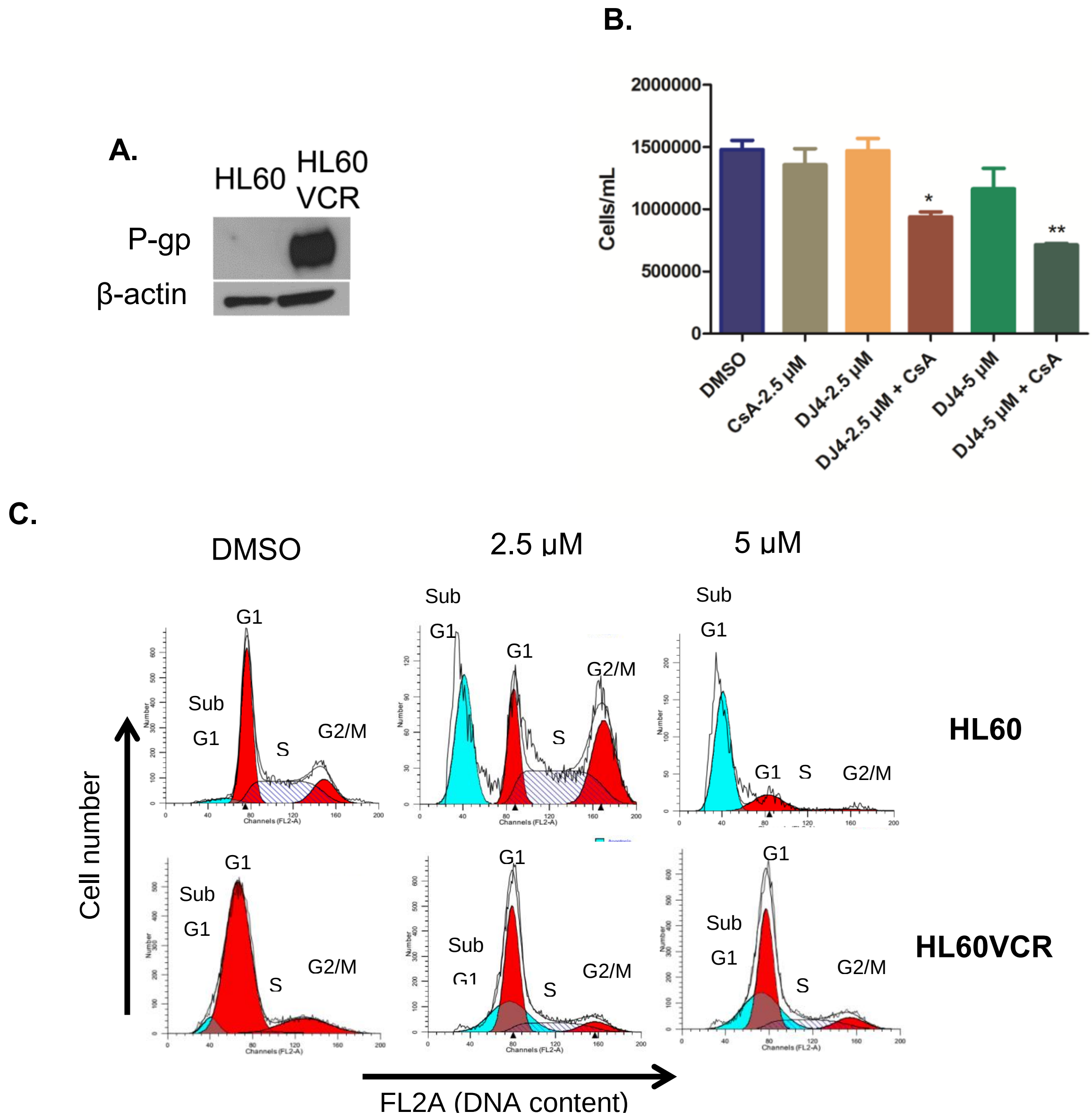

Figure S3. DJ4 was ineffective in multidrug resistant HL60VCR acute myeloid leukemia cells.

A. Endogenous expression of p-glycoprotein (P-gp) in HL60 and HL60VCR (multidrug resistant cells). β-Actin was used as internal control.

B. P-gp inhibitor cyclosporine A (CsA) treatment (2.5 μM) sensitized resistant cancer cells to DJ4. Cells were treated either with DJ4 or CsA alone or in combination. Number of cells were counted using Muse^{TM} cell analyzer. Statistical significance was analyzed by one-way ANOVA and Dunnett's multiple comparison post-test. P<0.05%. n=2. Data is representative of two independent experiments.

C. Cell cycle analysis in HL60 and HL60VCR cells. The cells were treated at 2.5 μM and 5 μM concentrations and analyzed using flow cytometry. Apoptotic cell population is indicated by sub-G1 phase.
